# Association of maternal polygenic risk scores for mental illness with perinatal risk factors for offspring mental illness

**DOI:** 10.1126/sciadv.abn3740

**Published:** 2022-12-14

**Authors:** Andrew Ratanatharathorn, Lori B. Chibnik, Karestan C. Koenen, Marc G. Weisskopf, Andrea L. Roberts

**Affiliations:** ^1^ Department of Epidemiology, Columbia University Mailman School of Public Health, New York, NY 10032, USA.; ^2^ Department of Epidemiology, Harvard T.H. Chan School of Public Health, Boston, MA 02115, USA.; ^3^ Department of Neurology, Massachusetts General Hospital, Boston, MA 02114, USA.; ^4^ Department of Social and Behavioral Sciences, Harvard T.H. Chan School of Public Health, Boston, MA 02115, USA.; ^5^ Broad Institute of MIT and Harvard, Cambridge, MA 02142, USA.; ^6^ Department of Psychiatry, Massachusetts General Hospital, Boston, MA 02114, USA.; ^7^ Department of Environmental Health, Harvard T.H. Chan School of Public Health, Boston, MA 02115, USA.

## Abstract

We examined whether genetic risk for mental illness is associated with known perinatal risk factors for offspring mental illness to determine whether gene-environmental correlation might account for the associations of perinatal factors with mental illness. Among 8983 women with 19,733 pregnancies, we found that genetic risk for mental illness was associated with any smoking during pregnancy [attention-deficit hyperactivity disorder (ADHD) and overall genetic risk], breast-feeding for less than 1 month (ADHD, depression, and overall genetic risk), experience of intimate partner violence in the year before the birth (depression and overall genetic risk), and pregestational overweight or obesity (bipolar disorder). These results indicate that genetic risk may partly account for the association between perinatal conditions and mental illness in offspring.

## Teaser

Maternal genetic risk for mental illness may cause offspring mental illness and perinatal factors of offspring mental illness.

## INTRODUCTION

Maternal factors, including alcohol and smoking during pregnancy (**1**–*9*), gestational diabetes, exposure to intimate partner violence (IPV) (*10*, *11*), pregestational weight (*12*–*15*), pregnancy length (*16*), preeclampsia (*17*, *18*), prior abortion, and the use of formula instead of breastfeeding (*19*), have been associated with offspring mental illness. The developmental origins of health and disease hypothesis posits that the intrauterine environment during gestation creates vulnerability to various health conditions later in life (*20*). Specifically relevant to mental health, intrauterine exposures may affect the development of brain structure and connectivity (*20*). For example, maternal nutrition affects birth weight, which has been associated with brain structure (*21*, *22*), thus potentially affecting risk for mental illness through this pathway (*20*).

**Fig. 1.**
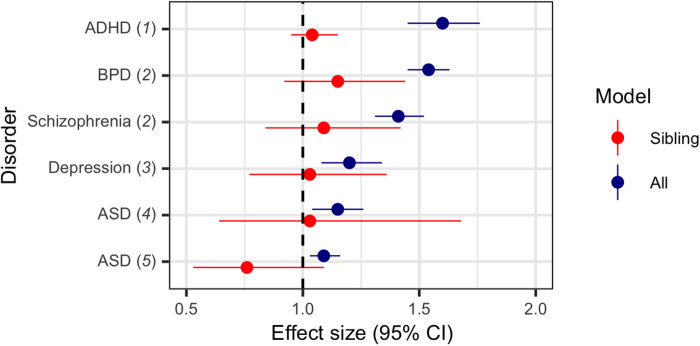
Previously reported attenuation of the association between maternal smoking and offspring risk of ADHD, bipolar disorder, schizophrenia, depression, and ASD from discordant sibling studies.

However, genetic risk for mental illness may confound the association between early life risk factors and mental illness in adulthood. For example, women with a greater genetic burden for attention-deficit hyperactivity disorder (ADHD) may be more likely both to smoke during pregnancy (*23*) and to pass on genetic loading for ADHD to their offspring, (*24*), which would create an association between maternal smoking and offspring ADHD. Studies of maternal smoking have found that significant associations with ADHD (*1*), autism spectrum disorder (ASD) (*4*, *5*), bipolar disorder (*2*), major depressive disorder (MDD) (*3*), and schizophrenia (*2*) have attenuated when using a discordant sibling design (see Fig. 1), indicating that genetic confounding or shared sibling environment may account for part of the observed association between maternal smoking during pregnancy and offspring mental illness. To examine whether genetic risk is a confounder, we tested whether genetic risk for six mental illnesses was associated with eight perinatal risk factors, using polygenic risk scores (PRS) derived from genetic data in the Nurses’ Health Study 2 (NHS2). For each participant, we calculated an overall genetic risk score by summing the six illness-specific PRS.

## MATERIALS AND METHODS

The NHS2 is an ongoing cohort of 116,430 female nurses recruited in 1989 and assessed every 2 years (*25*). Participants were 24 to 44 years old at baseline. Blood samples were collected from 29,611 participants between 1996 and 1999, as previously described (*26*). Genome-wide association study (GWAS) data were available for 13,313 women based on three genotyping platforms: (i) Illumina Human Hap Array (*N* = 781), (ii) Illumina Onco Array (*N* = 2722), and (iii) Illumina HumanCore Exome Chip (batch 1, *N* = 3276; batch 2, *N* = 4568). Participants with genetic data did not substantially differ from participants without genetic data across perinatal risk factors (table S4). Following a standard quality control pipeline (call rate > 0.90), participant genotype data were imputed using 1000 Genomes phase 3 reference data (*27*). Participants were restricted to those of European ancestry, given that PRS for mental illness were developed from GWAS of Europeans and may perform poorly for other ancestries, due to differences in linkage disequilibrium patterns and the frequency of minor alleles (*28*). Informed consent was received from all participants, and the study protocol was approved by the Institutional Review Boards of the Brigham and Women’s Hospital and the Harvard T.H. Chan School of Public Health.

### Polygenic risk scores

PRS for ADHD (*24*), ASD (*29*), BPD (*30*), MDD (*31*), neuroticism (*32*), and schizophrenia (*33*) were calculated using the summary statistics from the largest published GWAS, with *P* value thresholds, clumping parameters, minor allele frequencies, and imputation score cutoffs based on those found to explain maximum variance based on Nagelkerke’s *R*^2^ from each analysis (see table S1) (*25*, *34*, *35*). Participants’ PRS for each mental illness were calculated by taking the weighted sum of risk alleles, with each allele weighted by the log ORs reported in published GWAS summary statistics using PRSice-2 software (*36*, *37*). PRS were then standardized using *z*-score transformations. To investigate whether a nonspecific, overall risk for mental illness was associated with perinatal risk factors, we additionally created an overall PRS for each participant by summing the PRS for each of the six mental illnesses, which was then standardized using a *z*-score transformation.

### Perinatal risk factors

In 2001, participants were asked about each of their pregnancies that lasted 12 weeks or longer. The year the pregnancy ended, the outcome of the pregnancy (e.g., live birth or miscarriage) and whether the participant smoked cigarettes or drank alcohol during the pregnancy were queried, and, if so, the frequency (table S3). Participants reporting any alcohol or tobacco use were coded as having drunk or smoked during the pregnancy. Participants’ history of gestational diabetes and toxemia/preeclampsia during pregnancy was assessed starting in 1989 and updated biennially. Preterm delivery was defined as pregnancies shorter than 37 weeks, while those longer than 42 weeks were defined as postterm deliveries. Participants reported the birth weight of each child as <5.5 (2495 g), 5.5 to 9.9 (2496 to 4535 g), or ≥10 pounds (4536 g). Intimate partner abuse was assessed with four questions. Participants were asked whether they ever feared their partner, or were emotionally, physically, or sexually abused by their partner, and the year in which the abuse occurred. Participants who indicated that any of these events occurred in the calendar year before the birth year were considered perinatally exposed. Pregestational BMI (kilograms per square millimeter) was defined as BMI in the year before the child’s birth year, calculated from biennially reported weight and self-reported height in 1989. Participants were asked lifetime history of abortions and age at occurrence in 1993, 1997, 1999, and 2001. Lifetime history and age at occurrence of toxemia or preeclampsia during pregnancy and gestational diabetes were assessed in 1989 and updated biennially. In 1997, participants were asked for each pregnancy through then about whether they breastfed for more than 1 month.

### Covariates

We accounted for residual population stratification—systematic differences in allele frequencies across ancestries that can lead to spurious results—by including 10 principal components derived from the GWAS data as covariates (*25*, *38*).

### Statistical analyses

Power analyses were conducted a priori to estimate the minimum prevalence of a perinatal risk factor necessary to estimate an OR of 1.10 in our sample, and analyses were limited to the risk factors that met the prevalence threshold. Pearson correlations between each pair of PRS were estimated. To ascertain whether genetic risk for mental illness was associated with perinatal risk factors, we estimated ORs and 95% CIs of each outcome in association with a one SD increase in PRS score, using separate generalized estimating equations for each disorder and the overall PRS. To account for clustering by family, we used an independent correlation structure and either a logistic model for binary risk factors (e.g., IPV) or a multinomial logistic model for categorical risk factors (e.g., birth weight). All models were adjusted for genomic assay and 10 GWAS principal components. For each PRS, we calculated *P* values for each perinatal risk factor, which were then adjusted for multiple hypothesis testing using the false discovery rate method (*39*). All analyses were performed using R version 4.02.

## RESULTS

The prevalences of perinatal risk factors are reported in Table 1. Statistical power was adequate to identify an odds ratio (ORs) = 1.10 for perinatal factors with a prevalence of 5.5% in our data (fig. S1). As the prevalence of both high and low birth weight, gestational diabetes, and preeclampsia were below this threshold, they were excluded from our main results, although we report their associations with each PRS in table S2.

**Table 1.** Prevalence of perinatal risk factors in the NHSII.

	%	*N*
**Mothers**	–	8983
**Offspring**	–	19,733
**Any smoking during pregnancy**	10.6	2083
**Any alcohol use during pregnancy**	13.7	2703
**Breastfed < 1 month**	21.4	4213
**IPV**	11.9	2358
**Pregestational overweight/obese**	14.9	2934
**Prior abortion**	12.4	2444
**Pregnancy length**		
Postterm delivery (>42 weeks)	7	1388
Preterm delivery (<37 weeks)	6.9	1355
**Birth weight**		
<5.4 lbs. (2449 g)	3.2	624
>10 lbs. (4536 g)	2.6	515
**Gestational diabetes**	2.9	574
**Preeclampsia**	4.1	803

**Fig. 2.**
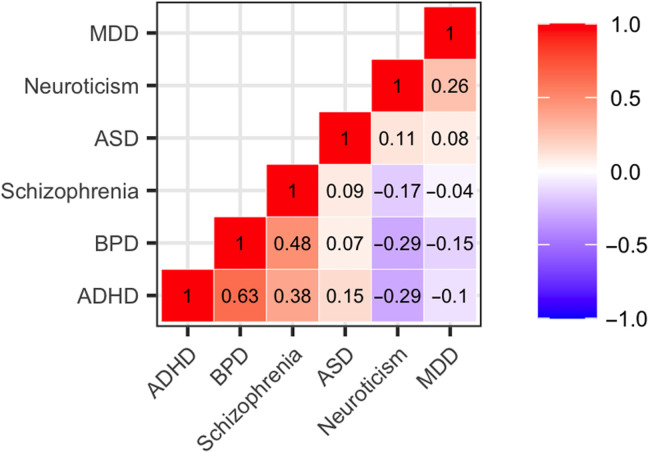
Correlations between PRS for ADHD, ASD, BPD, MDD, neuroticism, and schizophrenia.

Two distinct groups of PRS were found after examining correlations between PRS. ADHD, bipolar disorder (BPD), and schizophrenia were positively correlated with each other and negatively correlated with neuroticism and MDD, while ASD was uncorrelated with any other disorder (Fig. 2) (*25*).

**Fig. 3.**
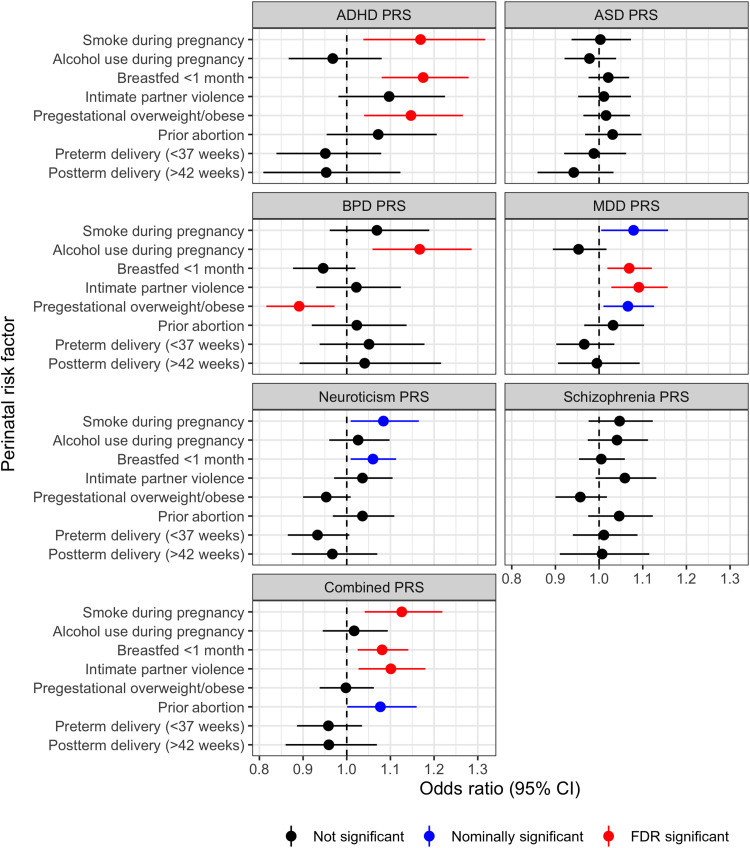
ORs and 95% CIs for each PRS on each perinatal outcome. Nominally significant (*P* < 0.05) associations are highlighted in blue, and those significant after false discovery rate (FDR) adjustment are highlighted in red.

Associations between each PRS and perinatal risk factor are presented in Fig. 3. After correction for multiple testing, a one SD increase in the PRS for ADHD was associated with being overweight/obese before pregnancy [OR: 1.15; 95% confidence interval (CI): 1.04 to 1.27], breastfeeding for <1 month (OR: 1.18; 95% CI: 1.08 to 1.28), and any smoking during pregnancy (OR: 1.17; 95% CI: 1.04 to 1.32). A one SD increase in the bipolar disorder PRS was associated with higher risk of alcohol use during pregnancy (OR: 1.17; 95% CI: 1.06 to 1.29) and lower likelihood of being overweight/obese before pregnancy (OR: 0.89; 95% CI: 0.82 to 0.97). Polygenic risk for major depression was associated with increased risk of experiencing IPV after adjusting for multiple testing (OR per SD: 1.09; 95% CI: 1.03 to 1.16) and nominally associated (i.e., without accounting for multiple testing) with being overweight/obese before pregnancy (OR per SD: 1.07; 95% CI: 1.01 to 1.13) and any smoking during pregnancy (OR per SD: 1.08; 95% CI: 1.00 to 1.16). Polygenic risk for neuroticism was also nominally associated with any smoking during pregnancy (OR: 1.09; 95% CI: 1.01 to 1.17). Genetic risk for ASD and schizophrenia was not associated with any perinatal risk factors. Higher overall genetic risk for any mental illness (combined PRS) was associated with any smoking during pregnancy (OR: 1.13; 95% CI: 1.04 to 1.22), breastfeeding less than 1 month (OR: 1.08; 95% CI: 1.03 to 1.14), and the experience of IPV (OR: 1.10; 95% CI: 1.03 to 1.18) after correction for multiple testing, and nominally associated with prior abortion (OR: 1.08; 95% CI: 1.00 to 1.16).

## DISCUSSION

We used molecular genetics to examine the association of maternal genetic risk for mental illnesses with perinatal risk factors for offspring mental illness. Genetic risk was associated with any smoking during pregnancy (ADHD), alcohol use during pregnancy (bipolar disorder), breastfeeding less than 1 month (ADHD and MDD), IPV (MDD), and risk of being overweight or obese before pregnancy (ADHD and bipolar disorder). No significant associations were found between genetic risk for ASD or schizophrenia and any perinatal factor. We found that combined PRS for mental illness was associated with any smoking during pregnancy, breastfeeding less than 1 month, IPV, and nominally with prior abortion, which was due to consistent positive associations with each of the individual PRS. Our results replicate and build upon those by Leppert *et al.* (*40*), which found that PRS for ADHD, but not PRS for ASD or schizophrenia, was associated with multiple perinatal risk factors, including any smoking during pregnancy and pregestational body mass index (BMI).

As PRS were associated only with exposures closely tied to maternal behavior (e.g., smoking, breastfeeding, and pregestational overweight or obesity), and not pregnancy length, these results raise the possibility that maternal genetic loading for mental illness may affect maternal behaviors, which then manifest as perinatal risk factors for offspring, who also inherit maternal genetic risk for mental illness. Similarities in the association of PRS with perinatal factors across disorders may reflect the convergence of shared factors across mental illnesses. For example, smoking initiation and relapse have been associated with negative affect (*41*, *42*), which is a factor shared by MDD, neuroticism, and ADHD. In addition, genetic risk for mental illness has been associated with risk-taking behavior (*43*, *44*), low self-esteem (*45*), deficits in emotional regulation (*46*), and reduced ability to interpret facial expressions (*47*, *48*). These traits are associated with increased risk of being targeted for IPV and might be pathways through which genetic risk is associated with the experience of IPV in the perinatal period (*49*–*52*).

Gene-environment correlation could also occur across generations if mental illness in the parental generation resulted in an adverse childhood environment for offspring, such as low socioeconomic status (*53*), divorce (*54*, *55*), or poor parenting (*56*, *57*), which could then increase risk of offspring experiencing IPV and offspring risk for mental illness through inherited genetic risk. Cross-generation gene-environmental correlations may also explain the association between PRS for mental illness and breastfeeding less than 1 month, as maternal ADHD has been associated with nonexclusive breastfeeding (*58*) and maternal depression with breast feeding less than 1 month (*59*).

Our study has at least four limitations. First, except for schizophrenia (Nagelkerke *R*^2^ = 0.184) (*33*), the PRS explain a small proportion of the variance in mental health outcomes (Nagelkerke *R*^2^ range: 0.01 to 0.05; table S1) (*25*). The use of PRS that explain little of the variation in mental illness may lead to attenuated estimates of the true associations between genetic risk and perinatal risk factors. In addition, while our results indicate that gene-environmental correlation may be responsible in part for the observed associations between perinatal risk factors and offspring mental illness, the low explanatory power of current mental illness PRS means that we cannot estimate the degree to which they confound these observed associations. Second, our sample is composed of parous nurses, a selected population with possibly little underlying risk for severe mental illness (e.g., schizophrenia), as onset of mental illness would disrupt the educational attainment necessary to enter the cohort. As a result, our estimates of genetic confounding are likely to be underestimated. Third, perinatal risk factors were self-reported, which may lead to underreporting of health behaviors believed to be harmful to the fetus, such as smoking during pregnancy. However, previous validation studies in the NHS2 have found participants to be reliable reporters of health behaviors and conditions such as BMI (*60*), ADHD (*61*), and eating habits (*62*). Fourth, residual population stratification could lead to spurious associations between the PRS and perinatal risk factors, especially given that the PRS explain a small proportion of variance in mental health outcomes (*63*).

In closing, our results indicate that genetic risk may account in part for previously identified associations between perinatal factors and offspring mental illness. These results warrant consideration when evaluating the degree to which interventions to reduce perinatal risk factors will affect offspring mental health.
