# Supplementary material for: Association of maternal polygenic risk scores for mental illness with perinatal risk factors for offspring mental illness
Source: Sci Adv. 2022 Dec 14;8(50):eabn3740. doi: 10.1126/sciadv.abn3740 (PMC9750139; doi:10.1126/sciadv.abn3740)
Supplement: Supplementary file 1 — Fig. S1 Tables S1 to S4 [file sciadv.abn3740_sm.pdf]

Science Advances
AAAS

Supplementary Materials for

Association of maternal polygenic risk scores for mental illness with perinatal risk factors for offspring mental illness

Andrew Ratanatharathorn *et al.*

Corresponding author: Andrew Ratanatharathorn, ar3054@columbia.edu

Sci. Adv. **8**, eabn3740 (2022)
DOI: 10.1126/sciadv.abn3740

This PDF file includes:

Fig. S1
Tables S1 to S4

Figure S1. Power analysis determining the minimum prevalence to identify an odds ratio (OR) with 80% power

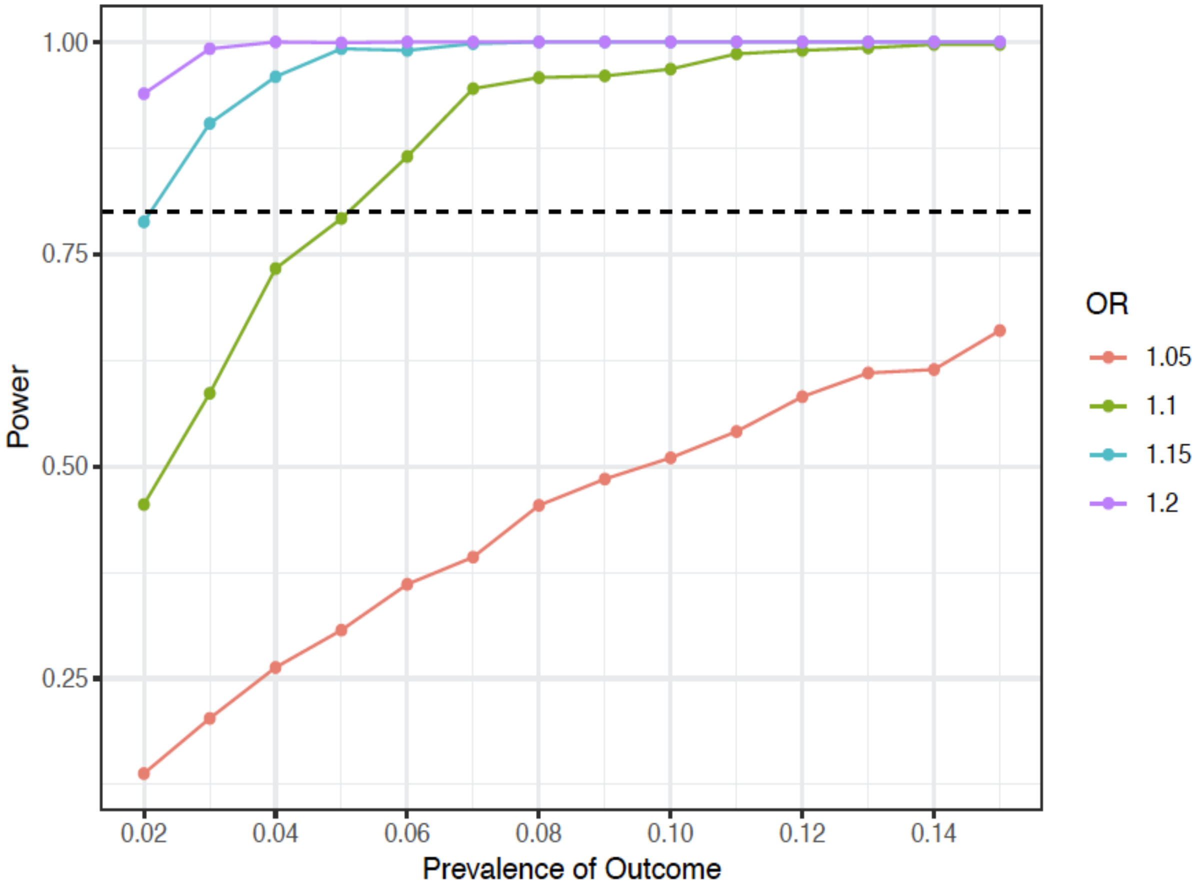

Table S1. Genome-wide association study summary statistics and parameters for polygenic risk scores (PRS) (*25*)

Disorder	Abbreviation	PRS p-value threshold	Nagelkerke's R2	MAF	INFO	Clumping r2	Clumping kb
Attention-Deficit/Hyperactivity Disorder (*24*)	ADHD	0.1	0.0103	0.01	0.8	>0.25	500
Autism Spectrum Disorder (*29*)	ASD	0.1	0.0245	0.05	0.9	>0.1	500
Bipolar Disorder (*30*)	BPD	0.01	0.04	0.05	0.9	>0.1	500
Major Depressive Disorder (*31*)	MDD	0.05	0.05	0.01	0.9	>0.1	500
Neuroticism (*32*)	Neuroticism	0.05	0.0279	0.01	0.9	0.25	250
Schizophrenia (*33*)	SCZ	0.05	0.184	-	-	-	-

Table S2. Odds ratios (95% confidence intervals) between PRS for each mental illness and perinatal risk factor

PRS	Prior Abortion	Alcohol use during Pregnancy	Gestational Diabetes	Smoke during Pregnancy	Preeclampsia	Intimate Partner Violence
ADHD	1.07 (0.95, 1.21)	0.97 (0.87, 1.08)	0.96 (0.77, 1.19)	1.17 (1.04, 1.32)	1.1 (0.95, 1.28)	1.1 (0.98, 1.23)
ASD	1.03 (0.97, 1.1)	0.98 (0.92, 1.04)	0.94 (0.84, 1.05)	1 (0.94, 1.07)	0.99 (0.91, 1.07)	1.01 (0.95, 1.07)
BPD	1.02 (0.92, 1.14)	1.17 (1.06, 1.29)	1.12 (0.93, 1.34)	1.07 (0.96, 1.19)	0.94 (0.82, 1.08)	1.02 (0.93, 1.12)
MDD	1.03 (0.97, 1.1)	0.95 (0.89, 1.02)	1.15 (1.03, 1.29)	1.08 (1, 1.16)	1.03 (0.95, 1.12)	1.09 (1.03, 1.16)
Neuroticism	1.04 (0.97, 1.11)	1.03 (0.96, 1.1)	1 (0.9, 1.12)	1.09 (1.01, 1.17)	0.99 (0.91, 1.08)	1.04 (0.97, 1.11)
SCZ	1.05 (0.98, 1.12)	1.04 (0.97, 1.11)	1.1 (0.97, 1.25)	1.05 (0.98, 1.12)	1.07 (0.97, 1.18)	1.06 (0.99, 1.13)
Combined	1.08 (1, 1.16)	1.02 (0.94, 1.1)	1.09 (0.96, 1.24)	1.13 (1.04, 1.22)	1.03 (0.94, 1.13)	1.1 (1.03, 1.18)

PRS	Pre-gestational Overweight/Obese	Breastfed <1 month	Birthweight: <5.4 lbs. (2,495 g)	Birthweight: >10 lbs. (4,536 g)	Post-term Delivery	Preterm Delivery
ADHD	1.15 (1.04, 1.27)	1.18 (1.08, 1.28)	0.92 (0.78, 1.1)	1 (0.8, 1.26)	0.95 (0.81, 1.12)	0.95 (0.84, 1.08)
ASD	1.02 (0.96, 1.07)	1.02 (0.98, 1.07)	1.03 (0.94, 1.13)	0.96 (0.85, 1.09)	0.94 (0.86, 1.03)	0.99 (0.92, 1.06)
BPD	0.89 (0.82, 0.97)	0.95 (0.88, 1.02)	1.03 (0.88, 1.2)	0.89 (0.72, 1.1)	1.04 (0.89, 1.22)	1.05 (0.94, 1.18)
MDD	1.07 (1.01, 1.13)	1.07 (1.02, 1.12)	1 (0.9, 1.1)	0.99 (0.87, 1.13)	1 (0.91, 1.09)	0.97 (0.9, 1.04)
Neuroticism	0.95 (0.9, 1.01)	1.06 (1.01, 1.11)	1 (0.91, 1.11)	0.95 (0.83, 1.09)	0.97 (0.87, 1.07)	0.93 (0.87, 1.01)
SCZ	0.96 (0.9, 1.02)	1.01 (0.95, 1.06)	0.99 (0.88, 1.1)	0.94 (0.81, 1.09)	1.01 (0.91, 1.12)	1.01 (0.94, 1.09)
Combined	1 (0.94, 1.06)	1.08 (1.03, 1.14)	1 (0.89, 1.12)	0.92 (0.78, 1.07)	0.96 (0.86, 1.07)	0.96 (0.89, 1.04)

Table S3. Self-reported drinks per week and cigarettes per day during pregnancy

Drinks per week					
**None**	**1**	**2-4**	**5-6**	**7-13**	**14+**
17030	2003	578	92	28	2
**Cigarettes per day**					
**None**	**<1**	**1-4**	**5-14**	**15-24**	**25+**
17651	374	372	613	587	136

Table S4. Prevalence of perinatal risk factors among NHS2 participants with and without genetic data

	N (%) without GWAS data	N (%) with GWAS data
**Smoking during Pregnancy**	10938 (10.8%)	2083 (10.6%)
**Alcohol use during Pregnancy**	13171 (13.0%)	2703 (13.7%)
**Breastfed <1 month**	23342 (26.0%)	4213 (22.4%)
**Intimate Partner Violence**	11670 (11.6%)	2358 (12.0%)
**Pre-gestational Overweight/Obese**	16749 (16.7%)	2934 (15.0%)
**Prior Abortion**	11676 (11.5%)	2444 (12.4%)
**Pregnancy length**		
Post-term Delivery (>42 weeks)	7426 (7.4%)	1388 (7.0%)
Preterm Delivery (<37 weeks)	7214 (7.1%)	1355 (6.9%)
**Birth Weight**		
<5.4 lbs. (2,449 g)	91675 (94.0%)	18032 (94.1%)
>10 lbs. (4,536 g)	2519 (2.6%)	515 (2.7%)
**Gestational Diabetes**	3372 (3.3%)	574 (2.9%)
**Preeclampsia**	4190 (4.1%)	803 (4.1%)
